# Multiphoton microscopy providing pathological-level quantification of myocardial fibrosis in transplanted human heart

**DOI:** 10.1007/s10103-022-03557-5

**Published:** 2022-04-08

**Authors:** Yuelong Yang, Liqin Zheng, Zhen Li, Jianhua Chen, Xinyi Wu, Guanmin Ren, Zebin Xiao, Xiaodan Li, Wei Luo, Zhigang Wu, Liming Nie, Jianxin Chen, Hui Liu

**Affiliations:** 1 The Second School of Clinical Medicine, Southern Medical University, Guangzhou, 510080 China; 2 Department of Radiology, Guangdong Provincial People’s Hospital, Guangdong Academy of Medical Sciences, Guangzhou, 510080 China; 3 Key Laboratory of OptoElectronic Science and Technology for Medicine of Ministry of Education, Fujian Provincial Key Laboratory of Photonics Technology, Fujian Normal University, Fuzhou, 350007 China; 4 Department of Radiology, The First Affiliated Hospital of Jinan University, Guangzhou, 510080 China; 5 Department of Pathology, Guangdong Provincial People’s Hospital, Guangdong Academy of Medical Sciences, Guangzhou, 510080 China; 6 Philips Healthcare China, Shenzhen, 518000, China; 7 Research Center of Medical Sciences and Department of Radiology, Guangdong Provincial People’s Hospital, Guangdong Academy of Medical Sciences, Guangzhou, 510080 China; 8 Guangdong Provincial Key Laboratory of Artificial Intelligence in Medical Image Analysis and Application, Guangdong Provincial People’s Hospital, Guangdong Academy of Medical Sciences, Guangzhou, 510080 China

**Keywords:** Multiphoton microscopy, Dilated cardiomyopathy, Ischemic cardiomyopathy, Myocardial fibrosis

## Abstract

Multiphoton microscopy (MPM), a high-resolution laser scanning technique, has been shown to provide detailed real-time information on fibrosis assessment in animal models. But the value of MPM in human histology, especially in heart tissue, has not been fully explored. We aimed to evaluate the association between myocardial fibrosis measured by MPM and that measured by histological staining in the transplanted human heart. One hundred and twenty samples of heart tissue were obtained from 20 patients consisting of 10 dilated cardiomyopathies (DCM) and 10 ischemic cardiomyopathies (ICM). MPM and picrosirius red staining were performed to quantify collagen volume fraction (CVF) in explanted hearts postoperatively. Cardiomyocyte and myocardial fibrosis could be clearly visualized by MPM. Although patients with ICM had significantly greater MPM-derived CVF than patients with DCM (25.33  ± 12.65 % vs. 19.82  ± 8.62 %, *p* = 0.006), there was a substantial overlap of CVF values between them. MPM-derived CVF was comparable to that derived from picrosirius red staining based on all samples (22.58 ± 11.13% vs. 21.19 ± 11.79%, *p* = 0.348), as well as in DCM samples and ICM samples. MPM-derived CVF was correlated strongly with the magnitude of staining-derived CVF in both all samples and DCM samples and ICM samples (r = 0.972, r = 0.963, r = 0.973, respectively; all *p* < 0.001). Intra- and inter-observer reproducibility for MPM-derived CVF and staining-derived CVF were 0.995, 0.989, 0.995, and 0.985, respectively. Our data demonstrated that MPM can provide a pathological-level assessment of myocardial microstructure in transplanted human heart.

**Supplementary Information:**

The online version contains supplementary material available at 10.1007/s10103-022-03557-5.

## Introduction

Abnormal deposition of myocardial fibrosis exacerbates the prognosis of many cardiac diseases, and the rising extent of fibrosis is associated with increased risk of cardiac and noncardiac outcomes [1–3]. Interstitial fibrosis and replacement fibrosis are two manifestations of myocardial fibrosis, which are characterized by the diffuse and focal accumulation of collagen [4]. The accurate characterization of collagen content is therefore an essential component in the pursuit of ultimate understanding of pathologies.

There are a few drawbacks of current methods for accurately detecting myocardial fibrosis in vivo and ex vivo. Although cardiac magnetic resonance (CMR) T1 mapping is a routine diagnostic tool for evaluating fibrosis content in vivo, this imaging technique suffers from low spatial resolution and fails to specifically evaluate the fibrosis content. Because mapping parameters of T1 value and extracellular volume fraction (ECV) may be affected by the deposition of other extracellular materials than collagen, for instance amyloid, iron et al. [5, 6]. For ex vivo specimens, tissue collagen is assessed by histochemistry, together with assistive technology such as immunohistochemistry or in situ hybridization. These standard methods require multiple steps of tissue processing and such sample preparation can lead to undesirable morphological alterations in cells and extracellular matrix [7].

Multiphoton microscopy (MPM) with the advantage of superior spatial resolution, high contrast, label-free imaging allows direct visualization of biological samples without invasive tissue staining [8]. Two-photon excitation fluorescence (TPEF) and second harmonic generation (SHG) microscopy are the two most commonly used multiphoton imaging modalities. Natural intrinsic fluorophores such as reduced nicotinamide adenine dinucleotide (NADH) and flavin adenine dinucleotides (FAD) are abundant in most cells and can emit strong TPEF signals [9]. Because of non-zero second-order generation susceptibility, fibrillar collagen possessing non-centrosymmetric structures are particularly strong SHG emitters [10]. Experiments conducted in several animal models have confirmed the ability of MPM to assess the microstructure and morphology in the liver tissue [11, 12]. However, histological staining validation of MPM imaging in human organs, especially in the heart, is lacking.

These features and distinct advantages of MPM motivated us to investigate the heart microstructures by MPM. Thus, the aims of the current study were (1) to evaluate the potential of MPM in visualizing cardiomyocyte and myocardial fibrosis; (2) to examine the relationship between MPM-derived collagen volume fraction (CVF) and staining-derived CVF measured by picrosirius red staining in the transplanted human heart.

## Materials and methods

### Study population

All research was carried out at Guangdong Provincial People’s Hospital between June 2020 and June 2021. Consecutive patients with ischemic cardiomyopathy (ICM) and dilated cardiomyopathy (DCM) undergoing heart transplant followed CMR were included in the study. The diagnosis of ICM and DCM was based on medical history, physical examination, electrocardiography, echocardiography, CMR, coronary angiography, and pathologic findings in reference to previously published criteria [13, 14]. A total of 20 patients constituted the study population. This study was approved by the Institutional Review Board of our hospital, and all patients gave informed consent conforming to the Declaration of Helsinki.

### Sample preparation

After each patient underwent heart transplantation, the explanted hearts were cut at basal, mid, and apical left ventricle (LV) levels and then fixed in 10% buffered formalin. Six tissue samples were taken from the mid-LV levels (6 segments) of each heart according to the American Heart Association 16-segment model before being embedded in paraffin. Two serial 5-μm thickness sections were cut from tissue samples for MPM imaging and the picrosirius red-staining, respectively. The digital images of the picrosirius red-stained sections were taken by a bright field light microscope (Pannoramic MIDI and Viewer, 3D Histech, Hungary).

### MPM image acquisition

MPM image acquisition was achieved using a previously described nonlinear optical imaging system [15]. Briefly, a commercial laser scanning microscope (LSM 880, Zeiss, Germany) equipped with a mode-locked femtosecond titanium (Ti): Sapphire laser (Chameleon Ultra, Coherent, USA) was used to obtain high-resolution images. The excitation wavelength (λex) used in this study was 810 nm. The backscattered signals were obtained via two independent channels at the same time: one channel for detecting SHG signal (green color) was set between 395 and 415 nm, the other channel for detecting TPEF signal (red color) was set between 428 and 677 nm. A Plan-Apochromat 20 × objective (NA = 0.8, Zeiss, Germany) and Plan-Apochromat 63 × oil objective (NA = 1.4, Zeiss, Germany) were employed for acquiring images from tissue samples. For the purpose of confirmation, a comparison of the MPM image to the picrosirius red-stained serial tissue slice was performed by an experienced pathologist.

### Image analysis

**Fig. 1:**
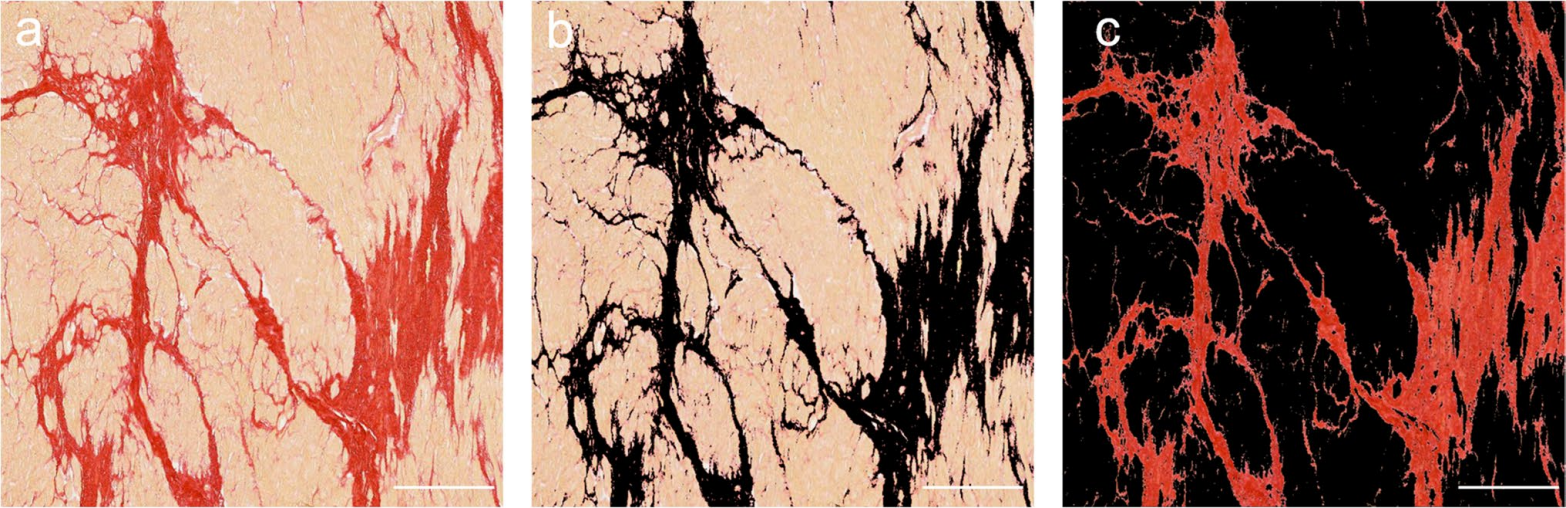
A diagram of quantitative collagen volume fraction (CVF) analysis from the picrosirius red-stained slice. The total myocardial area (**a**) and collagen area marked with black (**b**) were obtained semi-automatically using ImageJ software. Panel (**c**) shows the red collagen alone. The histological CVF was then calculated as the percentage of collagen area divided by the total myocardial area. Scale bars: 500 µm

The images obtained from picrosirius red-staining and MPM were analyzed through the ImageJ software (National Institute of Health, Bethesda, Maryland, USA). Collagen was separated from the myocardium by a color threshold plugin, and the collagen area was acquired from a combination of the standard deviations from mean signal and isodata automatic thresholding, as adopted by the previous studies [5]. As shown in Fig. 1, the collagen and myocyte components were stained red and yellow, respectively, and then the histological CVF was calculated as the percentage of collagen area divided by the total area of the sample.

### Statistical analysis

Continuous variables with a normal distribution based on the Kolmogorov–Smirnov test were presented as mean ± standard deviation (SD), and compared with the t-test. The data with a skewed distribution will be expressed as median and interquartile range (IQR) and compared using the Mann–Whitney U-test. Categorical variables were compared using the Chi-square test or Fisher exact test. Correlation between MPM-derived CVF and staining-derived CVF was assessed using Pearson’s correlation coefficients. The intra-observer and inter-observer reproducibility of the MPM-derived CVF and staining-derived CVF were assessed using Bland–Altman methods and intraclass correlation coefficient (ICC) in 20 randomly selected samples. A *p* value of < 0.05 on a two-sided test was considered statistically significant. All statistical analyses were performed with SPSS version 22.0 statistical software (SPSS, Chicago, Illinois, USA) and GraphPad Prism 6.0 (GraphPad Software, San Diego, California, USA).

## Results

### Clinical characteristics of the study population

The characteristics of the study population are outlined in Table 1. Of the 20 patients included in the study, 10 patients had ICM, while the rest had DCM. Patients with ICM were significantly higher in mean ages (53.8 ± 2.8 vs. 39.9 ± 4.8, *p* = 0.024) and high-sensitivity troponin T (224.5 [81.3–1274.0] pg/mL vs. 58.8 [19.5–176.0] pg/mL, *p* = 0.023) than patients with DCM. There were no significant differences in other clinical features and New York Heart Association class.

**Table 1:** Baseline characteristics according to the categories of cardiomyopathy

Variables	All patients (n = 20)	DCM (n = 10)	ICM (n = 10)	*p* value
Age (years)	46.9 ± 13.9	39.9 ± 4.8	53.8 ± 2.8	0.024
Male (n, %)	18 (90)	9 (90)	9 (90)	1.000
Height (cm)	167.5 ± 9.0	169.8 ± 7.8	165.1 ± 7.9	0.194
Weight (kg)	66.7 ± 13.0	71.8 ± 13.2	61.6 ± 11.3	0.077
BMI (kg/m^2^)	23.6 ± 3.8	24.8 ± 3.4	22.5 ± 4.1	0.197
BSA (m^2^)	1.7 ± 0.2	1.8 ± 0.2	1.7 ± 0.2	0.057
Heart rate (bpm)	78.8 ± 21.5	78.3 ± 21.7	79.2 ± 22.4	0.928
Smoking (n, %)	4 (20.0)	1 (10.0)	3 (30.0)	0.582
Hypertension (n, %)	7 (35.0)	3 (30.0)	4 (40.0)	1.000
Hyperlipemia (n, %)	1 (5.0)	0 (0.0)	1 (10.0)	1.000
Diabetes mellitus (n, %)	5 (25.0)	1 (10.0)	4 (40.0)	0.303
Family history of CAD (n, %)	1 (5.0)	0 (0.0)	1 (10.0)	1.000
NYHA Class III–IV (n, %)	15 (75.0)	8 (80.0)	7 (70.0)	1.000
NT-proBNP (pg/mL)	6733.9 ± 7193.9	7194.1 ± 9364.4	6273.6 ± 4592.6	0.783
hs-TnT (pg/mL)	84.7 (48.8–411.6)	58.8 (19.5–176.0)	224.5 (81.3–1274.0)	0.023

Data presented as mean ± SD, median (IQR) or n (%)

*DCM*, dilated cardiomyopathy; *ICM*, ischemic cardiomyopathy; *BMI*, body mass index; *BSA*, body surface area; *CAD*, coronary artery disease; *NYHA*, New York heart association; *NT-proBNP*, N-terminal pro-brain natriuretic peptide; *hs-TnT*, high-sensitivity troponin T

### MPM imaging

**Fig. 2:**
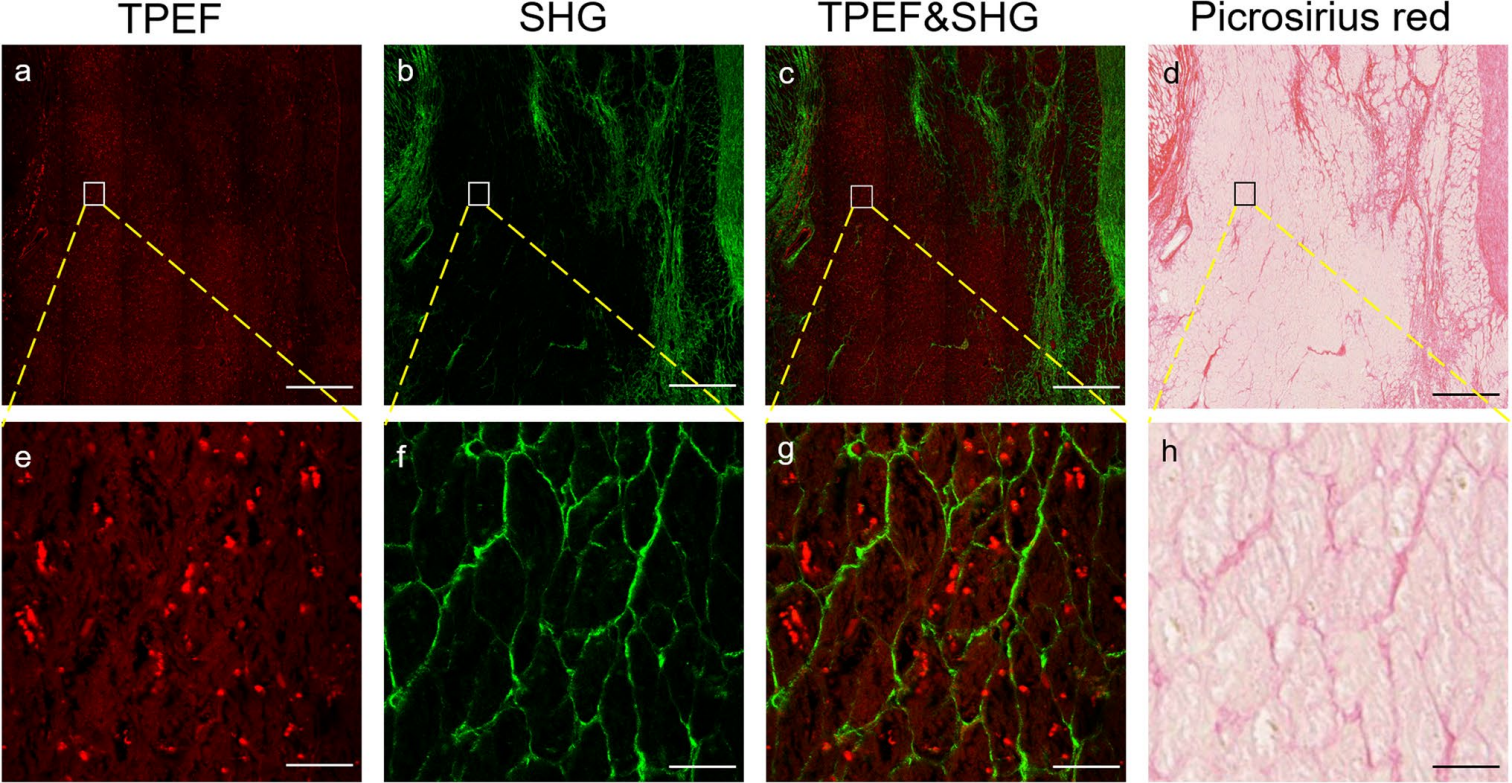
Visualization of myocardial microstructure. Examples of low-magnification (top, 20 ×) and high-magnification (bottom, 63 ×) images from MPM and picrosirius red staining. (**a**) TPEF image; (**b**) SHG image; (**c**) merging of SHG and TPEF images; (**d**) picrosirius red-stained image; (**e**–**h**) magnified MPM and picrosirius red-stained images of the white and black boxed regions, respectively. Cardiomyocyte (red) and myocardial fibrosis (green) could be clearly visualized by MPM. Scale bars: 500 µm in low-magnification images (top) and 25 µm in high-magnification images (bottom), MPM, multiphoton microscopy; TPEF, two-photon excited fluorescence; SHG, second-harmonic generation

Figure 2 shows the representative MPM images and the corresponding picrosirius red-stained images of ex vivo heart. The cardiomyocytes generate strong TPEF signals due to NADH and FAD in myocardial cells (Fig. 2a and e). The collagen fiber in myocardial interstitium delivers strong SHG signals because of their noncentrosymmetric molecular structure (Fig. 2b and f). The interaction between cardiomyocytes and interstitial fibrosis could be clearly visualized in TPEF&SHG images (Fig. 2c), and we can see them more clearly in a magnified image (Fig. 2g), whose details of cellular and interstitial organization intensively correlate with picrosirius red-stained images (Fig. 2d and h).

**Fig. 3:**
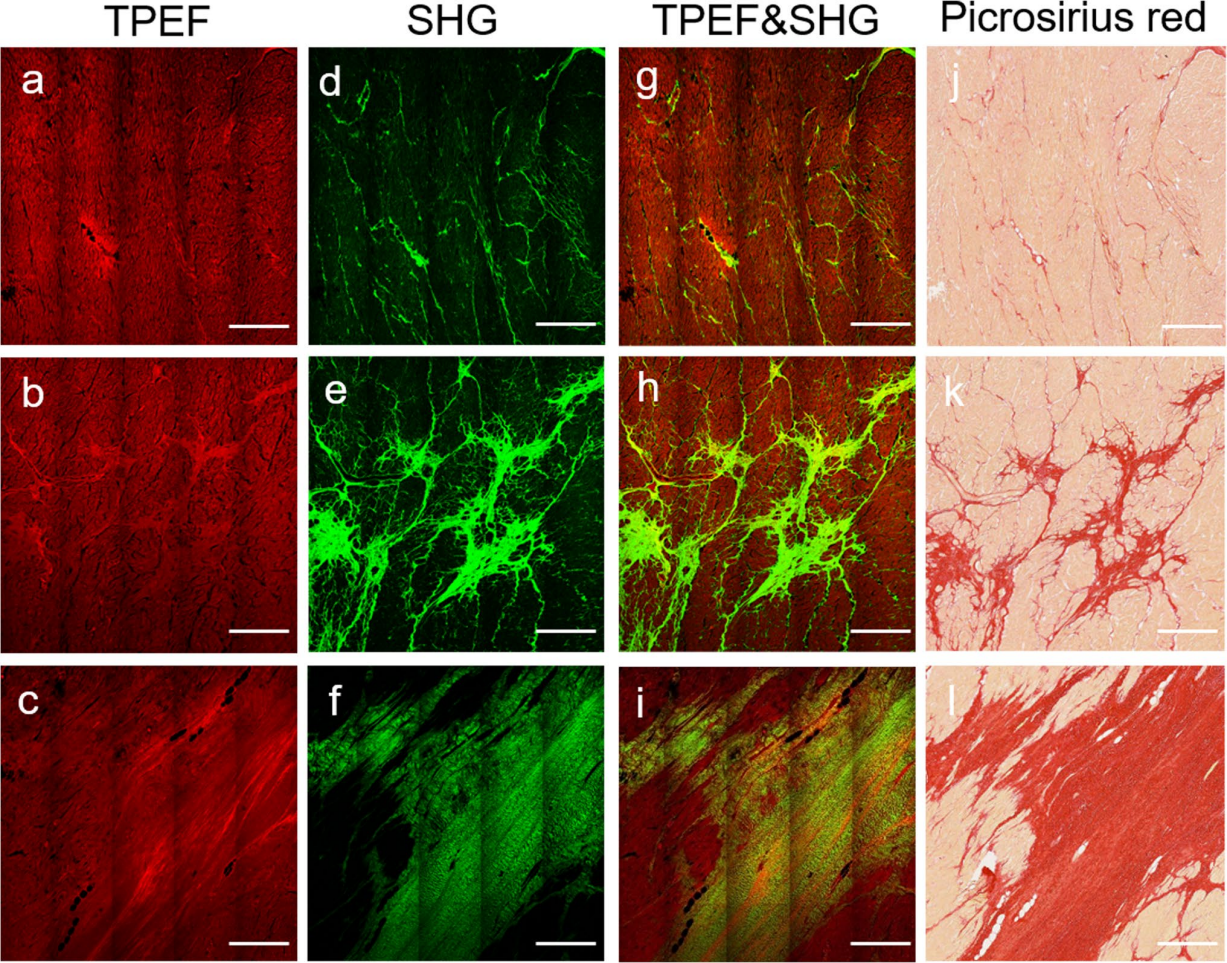
Representative images of different myocardial fibrosis types. (**a**–**c**) TPEF images; (**d**–**f**) SHG images; (**g**–**i**) merging of TPEF and SHG images; (**j**–**l**) corresponding images of picrosirius red staining. Examples shown in the figure correspond to interstitial fibrosis (top), a combination of interstitial and replacement fibrosis (middle), and replacement fibrosis (bottom), which reflect mild (10.92%), moderate (22.2%), extensive (54.51%) of MPM-derived CVF (**g**–**i**) and corresponding 9.82%, 21.4%, 57.56% of staining-derived CVF (**j**–**l**), respectively. MPM-derived CVF was comparable with staining-derived CVF. Scale bars: 500 µm for all images, CVF, collagen volume fraction; other abbreviation as in Fig. 2

Figure 3 shows the two types of myocardial fibrosis: interstitial and replacement fibrosis. Diffuse interstitial myocardial fibrosis was observed in 22/120 (18.3%) samples, and collagen fibers are scattered in the extracellular space (Fig. 3g). Focal replacement myocardial fibrosis was observed in 72/120 (60%) samples (Fig. 3i), which is local and occurs after cardiomyocyte necrosis. And the remaining 26/120 (21.7%) samples have both interstitial and focal replacement fibrosis (Fig. 3h). Three different manifestations from two types of myocardial fibrosis and their corresponding extent characterized by MPM are comparable with picrosirius red-stained images, respectively (Fig. 3j, l and k).

### Quantification of fibrosis

**Fig. 4:**
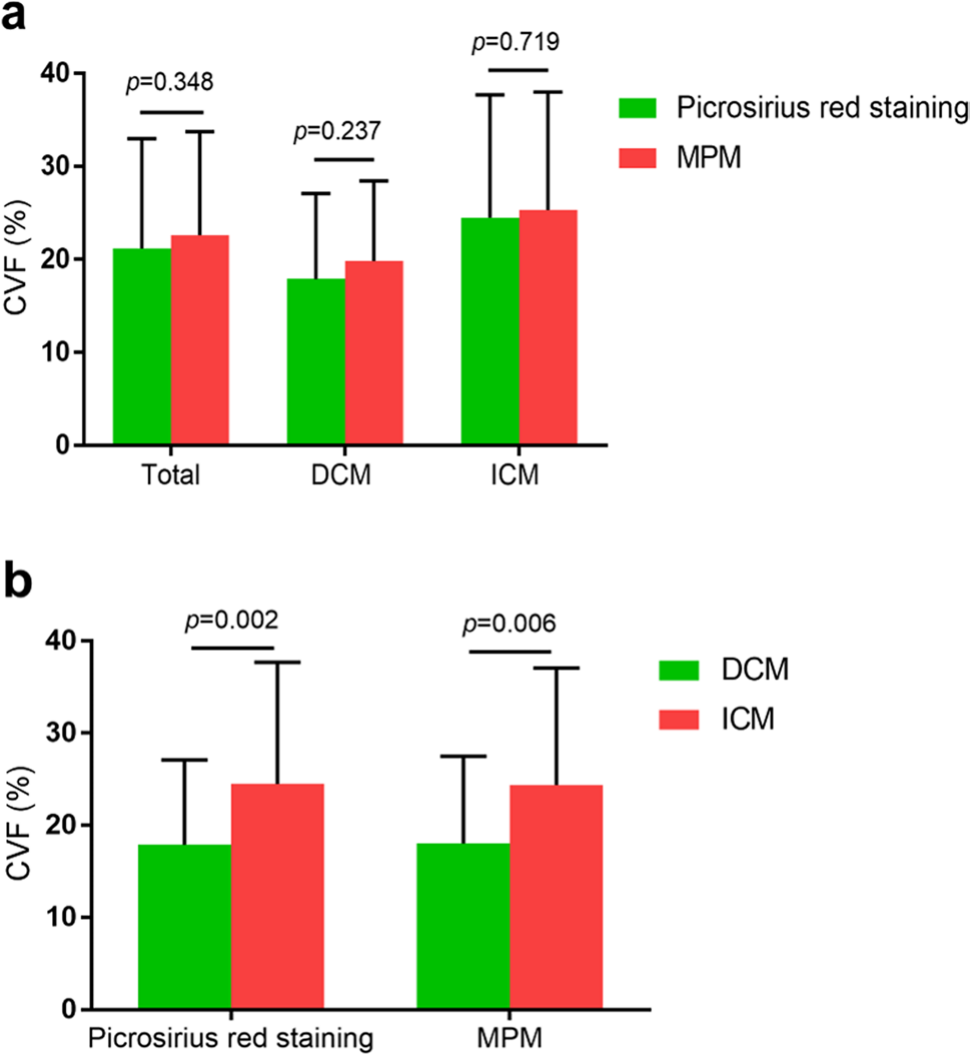
Comparisons of CVF based on different measurement modalities and cardiomyopathies. The CVF measured by MPM was comparable to that measured by picrosirius red staining, regardless of the type of cardiomyopathies (**a**). The CVF in ICM patients was slightly higher compared with that in DCM patients, regardless of the measurement modalities (**b**) ICM, ischemic cardiomyopathy; DCM, dilated cardiomyopathy; other abbreviation as in Fig. 3

The measurement of CVF was shown in Table 2 and Fig. 4. MPM-derived CVF was comparable to that derived from picrosirius red staining based on all samples (22.58 ± 11.13% vs. 21.19 ± 11.79%, *p* = 0.348), as well as in DCM samples and ICM samples (Fig. 4a). Patients with ICM had significantly greater MPM-derived CVF (25.33 ± 12.65% vs. 19.82 ± 8.62%, *p* = 0.006) and staining-derived CVF (24.48 ± 13.20% vs. 17.89 ± 9.18%, *p* = 0.002) than patients with DCM (Fig. 4b).

**Table 2:** Comparisons and correlations between MPM-derived CVF and picrosirius red staining-derived CVF

Cardiomyopathy (samples)	MPM-derived CVF (%)	Staining-derived CVF (%)	t value*	*p* value*	r value^#^	*p* value^#^
Total (n = 120)	22.58 ± 11.13	21.19 ± 11.79	− 0.941	0.348	0.972	< 0.001
DCM (n = 60)	19.82 ± 8.62	17.89 ± 9.18	− 1.189	0.237	0.963	< 0.001
ICM (n = 60)	25.33 ± 12.65	24.48 ± 13.20	− 0.361	0.719	0.973	< 0.001

Data presented as mean ± SD

^*^Comparisons between MPM-derived CVF and staining-derived CVF

^#^Correlations between MPM-derived CVF and staining-derived CVF

*MPM*, multiphoton microscopy; *CVF*, collagen volume fraction; *DCM*, dilated cardiomyopathy; *ICM*, ischemic cardiomyopathy

### Correlation between MPM-derived CVF and staining-derived CVF

**Fig. 5:**
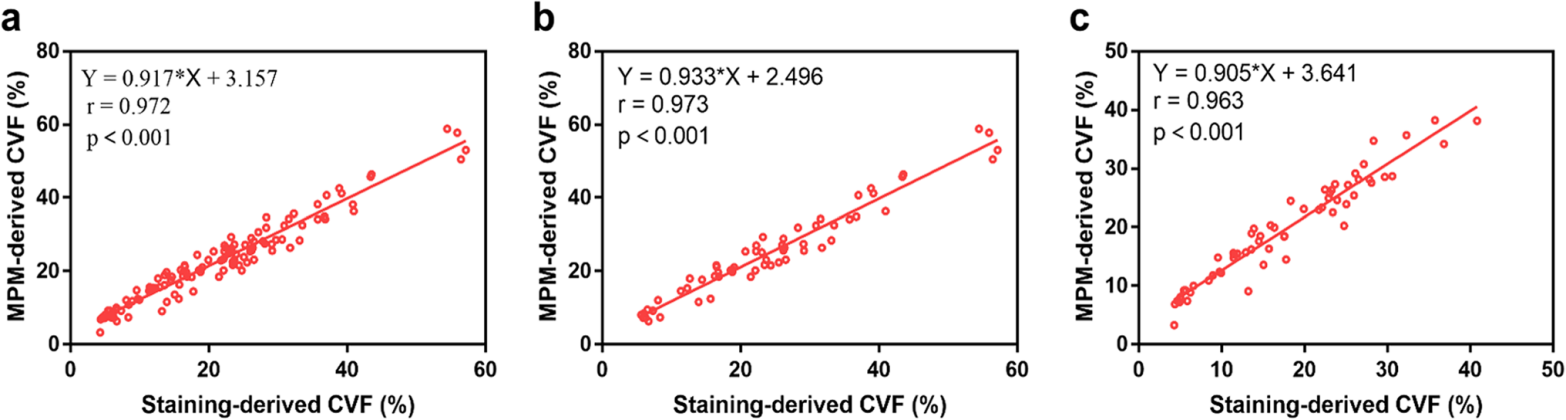
Correlations between MPM-derived CVF and staining-derived CVF. MPM-derived CVF correlated well with staining-derived CVF in all samples analysis of explanted hearts (**n = 120, a**). Based on subgroup analysis, correlations remained favorable in ICM samples (**n = 60, b**) and DCM samples (**n = 60, c**), Abbreviation as in Fig. 4

The results of the correlation between myocardial fibrosis measured by MPM and that measured by histological staining were shown in Table 2 and Fig. 5. Based on the analysis of all samples, the MPM-derived CVF values strongly correlated with the staining-derived CVF (r = 0.972, *p* < 0.001; Fig. 5a). Based on the subgroup analysis, the correlation between MPM-derived CVF and staining-derived CVF in ICM patients (r = 0.973, *p* < 0.001; Fig. 5b) was slightly higher compared with that in DCM patients (r = 0.963, *p* < 0.001; Fig. 5c).

### Reproducibility analysis

The intra- and inter-observer reproducibility was analyzed for the 20 samples (Table 3). MPM-derived CVF analysis revealed an ICC for intra-observer measurement of 0.995 (95% confidence interval [CI]: 0.987, 0.998) with a bias of − 0.3% (95% CI: − 2.8 to 2.1%) and for inter-observer measurement of 0.989 (95% CI: 0.971, 0.995) with a bias of − 1.1% (95% CI: − 4.8 to 2.5%). Staining-derived CVF analysis revealed an ICC for intra-observer measurement of 0.995 (95% CI: 0.988, 0.998) with a bias of − 1.1% (95% CI: − 3.3 to 1.1%) and for inter-observer measurement of 0.985 (95% CI: 0.962, 0.994) with a bias of − 1.0% (95% CI: − 4.9 to 2.9%) (as shown in the supplementary information).

**Table 3:** Intra- and inter-observer reproducibility showed by Bland–Altman analysis and intraclass correlation coefficient

		Bias	SD of bias	95% CI	ICC	95%CI
MPM-derived CVF (%)	Intra-observer variability	− 0.3	1.3	(− 2.8, 2.1)	0.995	(0.987, 0.998)
	Inter-observer variability	− 1.1	1.9	(− 4.8, 2.5)	0.989	(0.971, 0.995)
Staining-derived CVF (%)	Intra-observer variability	− 1.1	1.1	(− 3.3, 1.1)	0.995	(0.988, 0.998)
	Inter-observer variability	− 1.0	2.0	(− 4.9, 2.9)	0.985	(0.962, 0.994)

*SD*, standard deviation; *CI*, confidence interval; *ICC*, intraclass correlation coefficients; *CVF*, collagen volume fraction

## Discussion

The results of this study demonstrated that (1) MPM can readily visualize cardiomyocyte and myocardial fibrosis by TPEF and SHG microscopy; (2) the degree of CVF was higher in ICM patients than that in DCM patients; (3) the CVF measured using MPM was comparable to that derived from picrosirius red staining; (4) MPM-derived CVF correlated well with staining-derived CVF; and (5) both CVF measurements were reproducible.

We found that the visualization of myocardial microstructure is feasible in unstained histology sections using MPM. The change of myocardial microstructure involving cardiomyocyte and fibrosis damaged systolic or diastolic function [16]. The accurate estimation of these changes in various cardiomyopathies is a critical indicator for exploiting a therapeutic strategy and predicting prognosis. With heart biopsies, conventional histological analysis by suitable staining methods is regarded as the gold standard for the detection of myocardial microstructure [17]. But this process may be accompanied with dehydrated tissue, abnormal morphology, and time consuming. MPM imaging from unprocessed tissue with an advantage of minimal optical attenuation and photo-damage is a promising alternative for standard histopathological imaging to assess complex tissue microstructure. Our study demonstrated that myocardial fibrosis measured by MPM was almost equivalent to that measured by histological staining. Consequently, we have reason to believe that MPM could be used to explore the development process and mechanism of the disease, and also could serve as a complementary tool for the clinician to make a definite diagnosis and treatment monitoring of cardiovascular disease. To our knowledge, this is the first study that uses MPM to make an optical visualization of myocardial microstructure in the human heart.

We demonstrate whether it is diffuse and focal myocardial fibrosis, both of which can be accurately quantified using MPM. Focal fibrosis has been proven to represent the replacement of myocyte after cell damage or necrosis by fibrosis usually observed in ICM patients [18], different from diffuse interstitial myocardial fibrosis, which is scattered in the myocardial interstitium. Our findings show that the content of diffuse and focal myocardial fibrosis by MPM correlated well with that by picrosirius red staining, demonstrating that the ability of MPM to quantify fibrosis is not affected by fibrosis distribution. Because the intensity of the generated SHG signal depends intrinsically on the sample biological structure, and quantitative information can be obtained about the biomaterial structure and organization, compared with confocal microscopy, MPM can produce comparative high-axial and high-lateral resolution with added biochemical specificity [10].

In addition to the information with precise fibrosis distributions, MPM was used to detect the morphology of cardiomyocytes. Based on the selected MPM conditions (excitation 810 nm), no overlap of signals between SHG (395–415 nm emission) and TPEF (428–677 nm emission) images was observed in our experiment, which indicates the tissue specificity of the obtained signal. TPEF signals can be easily separated from SHG signals by using suitable filters, owing to the fact that TPEF microscopes based on fluorescent structures have different excitation mechanisms than SHG based on non-centrosymmetric molecules [19]. A similar MPM modality of 800 nm excitation has also been applied successfully to detect collagen and cell structures in ex vivo human liver specimens [20] and gastric cancer [21].

Currently, CMR is the most commonly used technique for the quantification of myocardial tissue characteristics in clinical practice. Although CMR-derived ECV has been clinically utilized as a surrogate for CVF in various heart diseases, it essentially represents histological extracellular space and does not necessarily have an excellent correlation with histological CVF. The correlation between ECV and CVF is highly variable ranging from 0.46 to 0.86 between different published studies [5, 22], which were inferior to the results of the present study with a stronger correlation of 0.97 between MPM-derived CVF and staining-derived CVF. Unfortunately, CMR has a spatial resolution of only 25–100 µm, which is so low that it did not allow us to characterize myocardial microstructure at the cellular level. MPM with a superior spatial resolution (1–1.6 µm) can compensate for this defect [23]. Despite limited imaging depth in comparison to full body for CMR, MPM can also meet the clinical need for the assessment of disease. Because most heart diseases, such as cardiomyopathy, myocarditis, myocardial amyloidosis, and so on, can lead to diffuse myocardial tissue-related changes, involving not only the middle but also the surface of myocardium layer.

The transformation of MPM imaging from subclinical to clinical stage is an unstoppable development trend. In terms of the current clinical application, MPM is mostly utilized in ex vivo tissues such as various tumor specimens in the early diagnosis and prognosis assessment [24, 25]. Real-time MPM of the human heart in vivo at cellular resolution is troublesome, the heart motion from the cardiac and respiratory cycles denotes a major challenge in studying complex biology in living systems. Along with advances in optical imaging instrumentation, the beating heart in the mouse has been successfully imaged using confocal and multiphoton imaging systems based on a combination of mechanical tissue stabilization and precise acquisition gating approaches [26]. The custom-built cardiac tissue stabilizer was used with direct contact in the epicardium to modulate and minimize gross motion, guaranteeing reproducibility in the slight motion over cardiac cycles during MPM imaging. Meanwhile, a prospective sequential cardiorespiratory gating scheme allows the image acquisition to be precisely synchronized with the cardiac cycle, which ensures that the imaging process is not affected by changes in physiological heart rate or rhythm. The reconstructed image without movement-related artifacts can be obtained by eliminating the phase between the image acquisition and the pacemaker signal and combining all consecutive levels of images at the same time points of the cardiac cycle [27, 28]. On the other hand, laser sources with photonic crystal fibers emitting around 1700 nm have been exploited particularly to image in deeper level [29]. Furthermore, updated and commercially available excitation sources match turn-key optical parametric amplifiers that support the research center to tune range from 1 to 2 µm [30]. With further developments in laser technology, a high repetition rate of optical sources in the mid-infrared range would facilitate clinical translation of MPM, which means performing non-destructive, in vivo evaluation of cardiac status such as disease severity and therapeutic efficacy.

### Study limitations

A few limitations in the present study were met. First, this was a single-center study, but the tissue sample size was large enough to validate the relationship between MPM imaging and histopathology. Second, MPM imaging was obtained in the ex vivo heart samples from the patients undergoing heart transplantation in this study, our next step is to perform a real-time intraoperative assessment of heart disease in vivo using high-resolution multiphoton microscopy.

## Conclusion

We demonstrated the feasibility of using MPM to assess accurately the morphology of cardiomyocyte and myocardial fibrosis in human ex vivo heart. MPM-derived CVF measured by MPM closely correlates with the staining-derived CVF. With the miniaturization and integration of probes, MPM has the potential to provide real-time in vivo imaging of the human heart at microscopic resolution.

## Supplementary Information

Below is the link to the electronic supplementary material.

Supplementary file1 (PDF 196 kb)
