# Supplementary material for: Multiphoton microscopy providing pathological-level quantification of myocardial fibrosis in transplanted human heart
Source: Lasers Med Sci. 2022 Apr 8;37(7):2889–98. doi: 10.1007/s10103-022-03557-5 (PMC9468057; doi:10.1007/s10103-022-03557-5)
Supplement: Supplementary file 1 — Supplementary file1 (PDF 196 kb) [file 10103_2022_3557_MOESM1_ESM.pdf]

Multiphoton microscopy providing pathological-level quantification of myocardial fibrosis in transplanted human heart

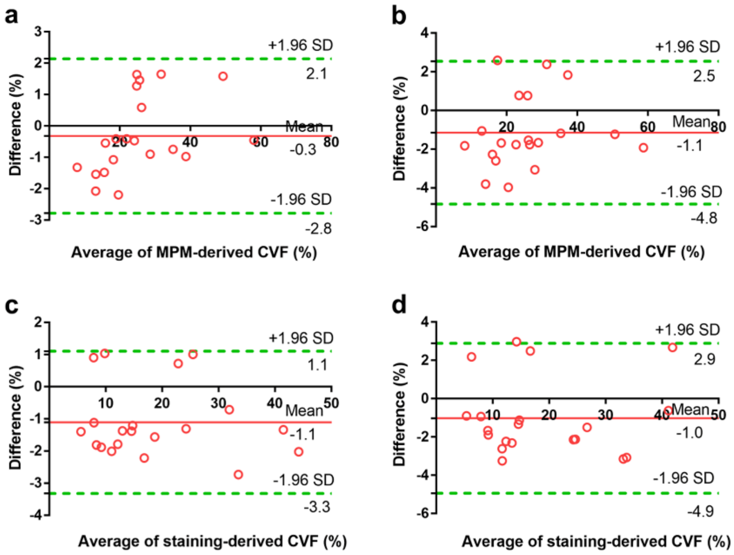

Supplemental Figure. Reproducibility of CVF in two measurement modalities. Bland-Altman shows inter- and intra-observer reproducibility of MPM-derived CVF (**a and b**) and staining-derived CVF (**c and d**)

CVF, collagen volume fraction; MPM, multiphoton microscopy
